# Isolated limb perfusion with biochemotherapy and oncolytic virotherapy combines with radiotherapy and surgery to overcome treatment resistance in an animal model of extremity soft tissue sarcoma

**DOI:** 10.1002/ijc.30162

**Published:** 2016-05-24

**Authors:** Michelle J. Wilkinson, Henry G. Smith, Timothy D. Pencavel, David C. Mansfield, Joan Kyula‐Currie, Aadil A. Khan, Gráinne McEntee, Victoria Roulstone, Andrew J. Hayes, Kevin J. Harrington

**Affiliations:** ^1^ Targeted Therapy TeamThe Institute of Cancer ResearchLondonUnited Kingdom; ^2^ Sarcoma/Melanoma Unit, Department Of Academic SurgeryThe Royal Marsden Hospital NHS Foundation TrustLondonUnited Kingdom

**Keywords:** oncolytic virotherapy, soft tissue sarcoma, isolated limb perfusion

## Abstract

The management of locally advanced or recurrent extremity sarcoma often necessitates multimodal therapy to preserve a limb, of which isolated limb perfusion (ILP) is a key component. However, with standard chemotherapeutic agents used in ILP, the duration of response is limited. Novel agents or treatment combinations are urgently needed to improve outcomes. Previous work in an animal model has demonstrated the efficacy of oncolytic virotherapy when delivered by ILP and, in this study, we report further improvements from combining ILP‐delivered oncolytic virotherapy with radiation and surgical resection. *In vitro*, the combination of radiation with an oncolytic vaccinia virus (GLV‐1h68) and melphalan demonstrated increased cytotoxicity in a panel of sarcoma cell lines. The effects were mediated through activation of the intrinsic apoptotic pathway. *In vivo*, combinations of radiation, oncolytic virotherapy and standard ILP resulted in delayed tumour growth and prolonged survival when compared with standard ILP alone. However, local disease control could only be secured when such treatment was combined with surgical resection, the timing of which was crucial in determining outcome. Combinations of oncolytic virotherapy with surgical resection and radiation have direct clinical relevance in extremity sarcoma and represent an exciting prospect for improving outcomes in this pathology.

## Abstract 2

**What's new?**

The management of locally advanced or recurrent extremity sarcoma often necessitates multimodal therapy to preserve a limb, of which isolated limb perfusion (ILP) is a key component. But the response to standard ILP is short‐lived in the majority of cases, making novel agents or treatment combinations urgently needed. Previous work in an animal model has demonstrated the effectiveness of combining oncolytic virotherapy with ILP. However, local disease progression was delayed rather than prevented. This paper demonstrates that durable local disease control can be achieved when ILP‐delivered oncolytic virotherapy is utilised as an induction therapy prior to surgical resection and radiotherapy.

The management of locally advanced or recurrent extremity soft tissue sarcoma (ESTS) is extremely challenging. As amputation has not been shown to improve survival, the goal of treatment in such cases is to secure local disease control and preserve limb function.^1^, ^2^, ^3^ Isolated limb perfusion (ILP) is a specialised surgical technique that forms a key component of the multimodal management of such patients. In the presence of a large, locally advanced ESTS that risks significant functional morbidity with resection, ILP may be used as a neoadjuvant therapy to downsize the tumour before surgical resection.^4^ If a tumour is truly inoperable by any means other than amputation, ILP is an alternative palliative option allowing symptom control and preservation of limb function.^5^

Using the standard protocol with melphalan and tumour necrosis factor α (TNF‐α), approximately 60% of patients have a significant response to ILP.^6^ Although some patients may benefit with long‐standing disease control, the response to ILP alone is short‐lived in the majority of cases with disease progression occurring within 12 months.^7^ Furthermore, the effects are limited to the perfusion field and have no significant impact on the dissemination of disease or overall survival.^8^ There is a clear need to improve outcomes in this challenging group of patients, either through the use of novel agents or the development of alternative treatment combinations.

With this aim in mind, an orthotopic model of ESTS and ILP has been developed.^9^ Oncolytic virotherapy was identified as a potential therapy for combination with ILP. Oncolytic virotherapy has been shown to be clinically effective when delivered by intra‐tumoural injection.^10^ However, the systemic administration of oncolytic virotherapy is hampered by sequestration within the reticulo‐endothelial system, clearance by circulating antibodies and an inability to penetrate the tumour in sufficient titres.^11^ Due to its ability to target the delivery of therapy directly to the tumour whilst affording protection from viral sequestration, ILP was thought to be ideally suited to combination with oncolytic virotherapy. This was confirmed in an *in vivo* model, where the addition of an oncolytic vaccinia virus (GLV‐1h68)^12^ to the standard ILP protocol, resulted in delayed tumour growth and prolonged survival.^9^ However, local disease control was not achieved and modifications to this treatment regimen were explored.

Radiotherapy has a well‐established role in securing local disease control following resection of ESTS.^2^, ^13^ Radiotherapy may also be used following an inadequate response to neoadjuvant ILP.^14^ Therefore, the combination of oncolytic virotherapy delivered by ILP and radiotherapy is a treatment regimen that may be readily translated into clinical practice. Furthermore, ionising radiation has been shown to be synergistic with oncolytic virotherapy in preclinical models of melanoma, glioma and head and neck cancers.^15^, ^16^, ^17^, ^18^, ^19^

In these studies, we investigated the efficacy of combining oncolytic virotherapy delivered by ILP with radiation and surgery to determine if this regimen can be exploited in the clinic to improve clinical outcomes in ESTS.

## Material and Methods

### *In vitro* studies

#### Cell lines

The BN175 rat sarcoma cell line was kindly donated by Prof. A Eggermont. This cell line is tumorigenic in Brown Norway rats.^20^ The CV1 monkey kidney cell line was obtained from existing laboratory stocks. The HT1080, SW684 and SW872 human sarcoma cell lines were donated by Dr. Janet Shipley. Cells were passaged in Dulbecco's Modified Eagle's Medium (DMEM), supplemented with 5–10% heat‐inactivated foetal bovine serum (FBS), 2.5% l‐glutamine and 1% penicillin/streptomycin. Cells were cultured at 37°C in an incubator maintaining a 10% carbon dioxide atmosphere.

#### Cytotoxic agents

GLV‐1h68 was produced and provided by Genelux Corporation (San Diego). GLV‐1h68 is an attenuated vaccinia virus strain and was constructed as previously described.^12^

Melphalan (Alkeran; Laboratoires Genopharm, France). Melphalan was supplied as a powder. Prior to injection it was dissolved in a diluent composed of water, sodium citrate, propylene glycol and ethanol, to a concentration of 1 mg/mL.

Recombinant human Tumour Necrosis Factor alpha was supplied as a lyophilised powder (First Link Ltd, Birmingham, UK) and was dissolved in PBS and stored at −20°C until use.

#### External beam radiotherapy

All *in vitro* and *in vivo* irradiations were performed using an orthovoltage X‐ray source (320/250 kV; serial no: 200090606; AGO X‐Ray Ltd, Reading, UK). The dose of radiation delivered was calculated with a universal dosimeter (UNIDOSE Universal Dosimeter, PTW, Grantham, UK).

#### LacZ detection

HT1080, SW684, SW872 and BN175 cells were plated at a density of 1 × 10^5^ per well in 24‐well plates. After incubation at 37°C for 16 hr, plates were treated with GLV‐1h68 at a MOI of 0.1. At specified time points, cells were fixed with 2% formaldehyde/0.2% glutaraldehyde then stained for 4 hr with X‐Gal staining buffer and X‐Gal (CalBioChem, Merck KGaA, Germany; 1:100) then washed with ultrafiltered water and dried.

#### Sulphorhodamine B assay

BN175 cells were plated at a density of 5 × 10^4^ cells per well in a 24‐well plate. After 16 hr cells were treated with either GLV‐1h68 MOI 0.01, melphalan 250 nM or both. After six hours, cells were irradiated at 0, 2, 4 or 8 Gy and then incubated at 37°C for 72 hr. Cell viability was quantified by fixing with 10% trichloroacetic acid (Sigma Aldrich, UK) and then staining with sulphorhodamine B (SRB; Sigma Aldrich, UK). The stained cells were dissolved with 1 mM TRIS (Sigma Aldrich, UK) and absorbance was measured at 570 nm on a plate reader (Victor 2, Perkin Elmer, MA).

#### Western blot analysis

Cells were plated at 5 × 10^5^ cells in 60 mm dishes. Following various treatments, cells were harvested after 48 hr in ice‐cold phosphate‐buffered saline (PBS), pelleted and resuspended in radioimmunoprecipitation assay buffer (50 mM Tris (pH 7.5), 150 mM NaCl, 1% NP40, 0.5% sodium deoxycholate and 0.1% SDS) with protease inhibitors (Roche Diagnostics Gmbh, Mannheim, Germany), 1 mM sodium orthovanadate (Sigma Aldrich, Gillingham, UK) and 10 mM sodium fluoride. Cells were then lysed by snap freezing on dry ice and allowed to thaw on ice for 10 min. The lysate was then centrifuged at 13,200 rpm at 4°C for 20 min to remove cell debris. Protein concentration was determined using the BCA protein assay (Pierce, Rockford, IL). Then 30 μg of each protein sample were resolved on SDS‐polyacrylamide gels (10–12%) and transferred to a polyvinylidene difluoride Hybond‐P membrane. Immunodetections were performed using procaspase and cleaved caspase 3 (Cell Signalling) rabbit polyclonal antibody in conjunction with a horseradish peroxidase (HRP)‐conjugated anti‐rabbit secondary antibody (GE Healthcare). Equal loading was assessed using glyceraldehyde‐3‐phosphate dehydrogenase‐GAPDH (Cell Signalling). The Super Signal chemiluminescent system (Pierce, Rockford, IL) or Immobilon Western chemiluminescent HRP substrate (Millipore) were used for detection.

#### Caspase‐Glo assay

The Caspase‐Glo 3/7 Assay (Promega) was used to determine the relative levels of Caspase 3/7 activation 48 hr postinfection with GLV‐1h68 (MOI 0.1), +/− melphalan 250 nM, +/− radiation (2 or 4 Gy) according to the manufacturer's instructions. Detected levels were normalised according to the proportion of surviving cells, as determined by duplicate MTT assay carried out simultaneously.

### *In vivo* studies

#### Animals

Inbred, specific pathogen‐free adult male Brown Norway rats weighing between 225 and 275 g were obtained from Charles River (Margate, UK) or Harlan (Nottingham, UK). They were housed in compliance with all relevant regulatory requirements and fed standard chow and water *ad libitum*.

#### Orthotopic model of advanced extremity sarcoma

The orthotopic model of extremity sarcoma was used as previously described.^9^ Tumour growth was assessed every 48 hr by direct calliper measurement in two orthogonal dimensions. Tumour volume was calculated using the formula: Volume = ½ (width^2^ × breadth). Animals were culled at an agreed humane endpoint of 2 cm maximum tumour diameter.

#### Isolated limb perfusion: Operative technique

**Figure 1:**
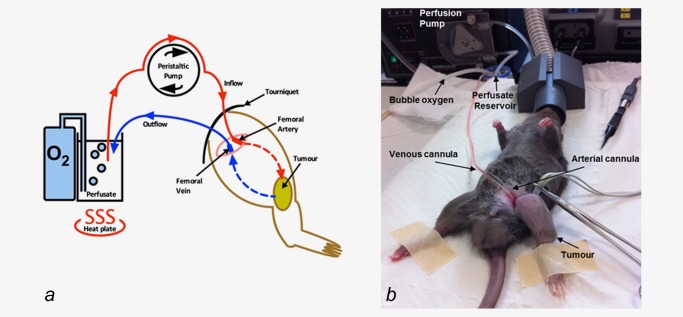
ILP perfusion circuit. (*a*) Cartoon schematic demonstrating the ILP perfusion circuit, using an oxygenated, hyperthermic perfusion reservoir into which the investigational therapeutic agents are added and (*b*) photograph of a Brown Norway rat undergoing ILP. [Color figure can be viewed in the online issue, which is available at wileyonlinelibrary.com.]

Cohorts were treated on Day 6 postengraftment of 1 × 10^7^ BN175 cells into the left hind limb, with a median tumour size of 1.4 cm^3^. The control group underwent a surgical procedure under general anaesthetic with open ligation of the left superficial femoral artery and vein using 5/0 ethilon. For the treatment cohorts, an ILP was performed on Day 6 as previously described.^9^ In brief, under general anaesthesia, a 1‐cm incision was made over the left groin crease. Using an operative microscope, the femoral vessels, nerve and inguinal ligament were visualised and exposed. The superficial femoral artery and vein were ligated proximally and controlled distally using 5/0 ethilon. A venotomy was then made and the vein cannulated. Twenty units of heparin in 2 mL of 0.9% saline were then injected into the vein allowing systemic circulation and anticoagulation. An arteriotomy was then made and the artery cannulated and connected to a perfusion pump using silicone tubing primed with 0.9% saline. An elastic band was used as a tourniquet, applied tightly to the left hind limb proximal to the cannulation site. The perfusion circuit was brought into continuity with the perfusate reservoir (containing 8 mL haemaccel, bubble oxygen and heated to 39°C) and a perfusion established through the hind limb at a flow rate of 4 mL/min. Once a stable perfusion was confirmed the required therapeutics were added to the perfusate (GLV‐1h68 1 × 10^7^ pfu, melphalan 100 μg, TNF‐α 50 μg) and the limb perfused for 13 min. The circuit was then washed out using 0.9% saline for 2 min. The tourniquet was then released and the artery and vein ligated distal to the cannulation site. The skin was then closed with 6/0 vicryl rapide and the operative site cleaned (Figs. 1a and 1b).

#### External beam radiotherapy

Therapeutic irradiations were performed under general anaesthesia using an intraperitoneal injection of 1.5–3 mL/kg of a 1:1:2 solution of Hypnorm (Janssen Pharmaceutical Ltd., UK), Hypnovel (Roche, UK) and sterile water. Anaesthetised animals were placed in a custom‐made 6 mm lead shield. The shield abducts the leg from the midline, such that the tumour may be irradiated through a window, whilst minimising irradiation of the surgical wound, abdominal viscera or other structures.

#### Surgical resection of tumours

Two therapeutic regimens combining ILP (melphalan, TNF‐α and GLV‐1h68) with irradiation (13 Gy in 2 fractions) and surgical resection were used. At either Day 3 or Day 10 postILP, surgical resection of the tumour was carried out under general anaesthetic. An elliptical incision was made in the skin over the palpable surface of the tumour. Adherent subcutaneous tissue and fascia was resected en‐bloc with the tumour. All visible tumour was removed as a single specimen without tumour rupture. At the deep margin of the tumour, the fibula and peroneal bundle were preserved to maintain limb function. The fascia and skin were closed in separate layers with 6/0 vicryl rapide.

### Statistical analysis

Simple and descriptive analyses were performed using Microsoft Excel (Microsoft Inc, WA). Kaplan–Meier survival analysis and group analysis, including ANOVA, were performed using GraphPad Prism software (GraphPad Software Inc, CA). Statistical significance was defined as a *p* values of less than 0.05. Error bars on all graphs represent standard error of the mean.

## Results

### GLV‐1h68 readily infects and replicates within rodent and human sarcoma cell lines

**Figure 2:**
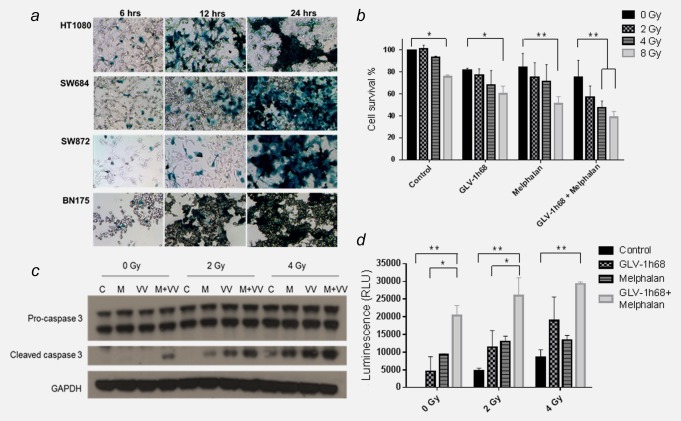
The combination of GLV‐1h68, melphalan and radiotherapy activates the effector caspase pathway and significantly increases cytotoxicity. (*a*) Microscopic image of GLV‐1h68 infection and LacZ transgene expression in a panel of rat and human sarcoma cell lines at increasing time points postinfection with GLV‐1h68 (MOI 1). (*b*) SRB assay showing increased cytotoxicity with triple combination therapy at 72 hr post‐treatment with GLV‐1h68 (MOI 0.01), melphalan (250 nM) and EBRT. This is statistically significant between 0 and 8 Gy for the control, GLV‐1h68 and melphalan‐treated cells and between 0 to 4 Gy and 0–8 Gy in the combination therapy (GLV‐1h68 and melphalan) cohort. (*c*) Induction of caspase 3 cleavage on western blot analysis and (*d*) Caspase–Glo assay showing quantitative analysis of caspase 3 and 7 production at 48 hr post‐treatment. With 0 and 2 Gy of EBRT, this is statistically significant between the control and melphalan and the control and combination therapy (GLV‐1h68 and melphalan) group. At 4 Gy statistical significance is only seen between the control and combination therapy group. (C = control, M = melphalan and VV = GLV‐1h68) (* = *p* values <0.01, ** = *p* values <0.01 and ** = *p* values <0.01). [Color figure can be viewed in the online issue, which is available at wileyonlinelibrary.com.]

The ability of GLV‐1h68 to infect and replicate within sarcoma cell lines was investigated by observing the number of cells expressing the GLV‐1h68 LacZ transgene product, β‐galactosidase, at successive time‐points. The BN175 rat fibrosarcoma cell line and a panel of human sarcoma cell lines comprising SW872 (liposarcoma), SW684 (fibrosarcoma) and HT1080 (fibrosarcoma) were tested. At 6, 12 and 24 hr post‐treatment with GLV 1h68 (MOI 1.0), cells were fixed and then stained for β‐galactosidase. Viral transgene expression was detectable as early as 6 hr postinfection in all cell lines. LacZ expression increased over the observed time period with almost complete infection of all cells, in all cell lines by 24 hr (Fig. 2 *a*).

### The cytotoxic effect of combination therapy with GLV‐1h68 and melphalan is significantly enhanced with the addition of ionizing radiation

The cytotoxic effects of combination therapy with GLV‐1h68 (MOI 0.1), melphalan (250 nM) and external beam radiotherapy (EBRT; 2, 4 and 8 Gy) were investigated using an SRB assay. When compared with either modality alone, the addition of radiation to melphalan or GLV‐1h68 only increased cytotoxicity at a dose of 8 Gy. A statistically significant increase in cytotoxicity was demonstrated with triple therapy compared to GLV‐1h68 combined with melphalan alone at doses of 4 and 8 Gy (Two‐way ANOVA with Bonferroni post‐test; *p* < 0.01; Fig. 2 *b*).

### Treatment with GLV‐1h68, melphalan and EBRT results in caspase‐3 cleavage and apoptotic cell death

At 48 hr, caspase cleavage was not induced by single agent therapy with GLV‐1h68 or melphalan. Low levels of caspase cleavage were induced by EBRT alone at a dose of 4 Gy. However, the combinations of melphalan and GLV‐1h68, GLV‐1h68 and EBRT or melphalan and EBRT resulted in greater cleavage of caspase‐3. This was markedly increased with triple combination therapy (GLV‐1h68, melphalan and EBRT) compared to double therapy at both 2 and 4 Gy (Fig. 2 *c*).

This increase in effector caspase‐3 and ‐7 was confirmed and quantified using a Caspase Glo assay. The combination of GLV‐1h68 and melphalan caused a significant increase in cleaved caspase 3/7 activity compared to controls at all doses of radiation (zero, 2 and 4 Gy; *p* < 0.01) and was superior to GLV‐1h68 alone and GLV‐1h68 with 2 Gy (two‐way ANOVA with Bonferroni post‐test; *p* < 0.05). These results suggest the primary mechanism of cell death is mediated through the apoptotic signalling pathway (Fig. 2 *d*).

### The addition of GLV‐1h68 and EBRT to standard ILP delays tumour growth and prolongs survival *in vivo*

The therapeutic efficacy of a standard ILP (melphalan and TNF‐α), triple therapy ILP (melphalan, TNF‐α and GLV‐1h68) and their combinations with EBRT (13 Gy in 2 fractions) was studied.

**Figure 3:**
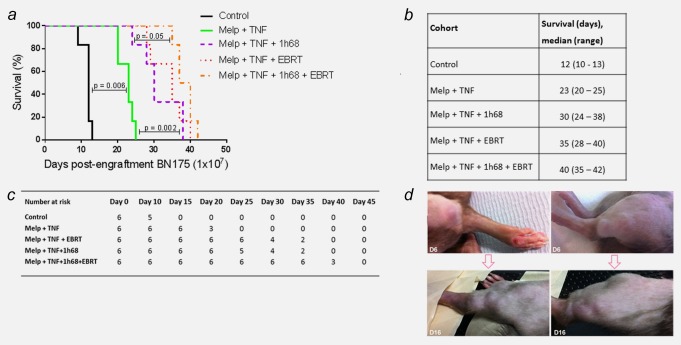
The addition of GLV‐1h68 and EBRT to standard ILP delays tumour growth and time to humane end‐point. Survival and tumour growth of animals, bearing BN175 tumours on the left hind limb, after differing treatment regimens. (*a*) Kaplan–Meier curve of treatment cohorts; time to humane endpoint. (*b*) Table of median time to humane endpoint in each cohort. (*c*) Table displaying surviving number of animals in each cohort at progressive time points. (*d*) Photographs of two separate animals showing maximum response to therapy at Day 6 post‐treatment with ILP (melphalan, TNF + GLV‐1h68) + EBRT and multifocal tumour relapse at day 16. [Color figure can be viewed in the online issue, which is available at wileyonlinelibrary.com.]

The addition of EBRT after standard ILP therapy resulted in an increase in median survival from 12 days for controls and 23 days for ILP alone to 35 days. This was highly statistically significant (Log‐rank (Mantel‐Cox) test; controls, *p* = 0.0005; standard ILP, *p* = 0.0006). The addition of EBRT to triple therapy ILP (melphalan, TNF‐α and GLV‐1h68) resulted in the greatest increase in survival. A significant increase in survival was achieved compared to standard ILP, from a median of 23–40 days (Log‐rank (Mantel–Cox) test; *p* < 0.0001). However, when compared to the combination of EBRT and standard ILP or triple therapy ILP, no significant increase in survival was seen. No increase in morbidity or mortality was seen in any of the treatment groups (Figs. 3 *a*–3 *c*).

On visual assessment of the limb and quantitative external calliper measurement of tumour growth, a consistent maximum response to therapy was seen at Day 6 postcompletion of treatment (Day 15 postengraftment of BN175). This response persisted for a maximum of 10 days prior to the consistent development of multifocal tumour relapse in the limb (Fig. 3 *d*).

### The timing of surgical resection is critical in producing a curative treatment regimen when combined with triple therapy ILP and EBRT

**Figure 4:**
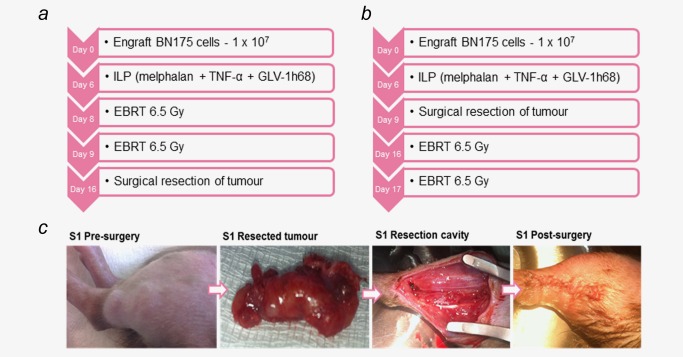
(*a* & *b*) Treatment regimens tested for ILP (melphalan, TNF‐α and GLV‐1h68), EBRT (13 Gy/2 fractions) and surgical resection. Tumours were measured by external caliper assessment every 2–3 days and the animals culled once tumours reached the maximum limit of 2 cm. (*c*) Photographs of an animal (S1), undergoing a marginal tumour resection according to the regimen described in 4A. [Color figure can be viewed in the online issue, which is available at wileyonlinelibrary.com.]

In an attempt to create a curative treatment regimen, two different treatment protocols combining triple therapy ILP with EBRT and surgical resection were developed (Figs. 4 *a* and 4 *b*). In the first protocol, the tumour was resected at the point of maximal response to ILP and EBRT on Day 16 postimplantation. The second regimen was developed to reflect clinical practice. ILP is often used as induction chemotherapy to downsize locally advanced tumours and may be followed by adjuvant radiotherapy to compromised margins. In this protocol, the tumour was resected following ILP on Day 9, with EBRT given one week later.

**Figure 5:**
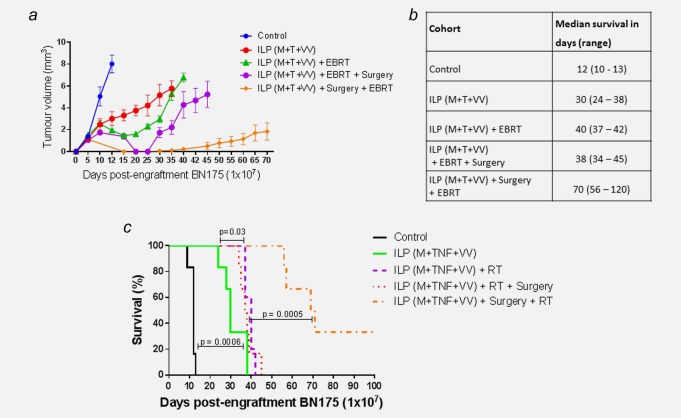
The combination of ILP with melphalan (M), TNF (T) and GLV‐1h68 (VV) as neoadjuvant chemotherapy prior to surgical resection and adjuvant EBRT significantly delays tumour growth and time to humane end‐point. (*a*) Tumour growth delay curve of Brown Norway rats, bearing BN175 tumours on the left hind limb, after differing treatment regimens. (*b*) Table of median time to humane endpoint in each cohort. (*c*) Kaplan–Meier curve of treatment cohorts; time to humane endpoint. [Color figure can be viewed in the online issue, which is available at wileyonlinelibrary.com.]

In the first regimen, local disease was controlled for 12 days in 5 of 6 animals and for 21 days in the final animal. All 6 animals developed local disease recurrence. An ulcerative recurrence developed rapidly in 3 animals, requiring the animals to be culled between 34–45 days postengraftment prior to reaching the maximum tumour limit of 2 cm (Figs. 5 *a*–5 *c*).

In comparison to triple therapy ILP with EBRT, the addition of postEBRT surgical resection did not increase survival, with median survivals of 40 and 38 days, respectively (Log‐rank (Mantel–Cox) test; *p* = 0.612).

**Figure 6:**
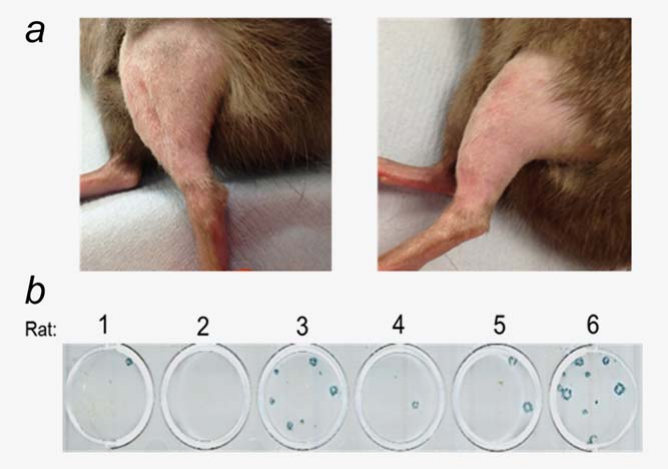
(*a*) Photographs of the treated limb in the two cured animals with no evidence of local or systemic recurrence at 120 days post‐tumour engraftment and treatment with ILP (melphalan + TNF‐α + GLV‐1h68) as induction chemotherapy followed by surgical resection and adjuvant EBRT. (*b*) Recovery of GLV‐1h68 in tumours 72 hr after ILP. Photograph of plate with viral plaque assay showing LacZ‐expressing live replication‐competent virus recovered from the tumours in five out of the six rats treated with triple therapy ILP (melphalan, TNF‐α and GLV‐1h68) 72 hr before tumour resection. Rat 1 and 3 remained disease free at 120 days postengraftment of tumour. [Color figure can be viewed in the online issue, which is available at wileyonlinelibrary.com.]

In the second regimen involving early surgery, a significant increase in survival was noted compared to the first regimen, with a median survival of 70 days (Log‐rank (Mantel–Cox) test; *p* < 0.001) (Figs. 5 *–c*). Furthermore, 2 of the 6 animals in this group were cured, with no evidence of local or systemic disease at Day 120 postengraftment of tumour (Fig. 6 *a*). Following resection, the tumours from all 6 animals were snap‐frozen and homogenised. Viral plaque assays were used to determine the presence of GLV‐1h68. Of the six tumours, five were found to have recoverable, replication‐competent virus still present at 72 hr after delivery of virus by ILP (Fig. 6 *b*).

### Local disease control is followed by systemic relapse

Three of the animals in the triple therapy ILP and EBRT cohort were culled before reaching their humane endpoint due to lethargy and respiratory distress. At autopsy, macroscopic lung metastases were found in all three animals. One animal also demonstrated inguinal and para‐aortic lymph node metastases. Cell lines were established from the lung metastases (LM) and para‐aortic lymph node (PN). Cell proliferation MTT assays were carried out to define treatment sensitivities of these metastatic cell lines to GLV‐1h68 and melphalan. No significant difference in sensitivity to these modalities was noted when compared to the parental BN175 cell line (Supporting Information Fig. 1 *a* and *b*). Using clonogenic assays the sensitivity of the metastatic cell lines to radiation was assessed. Interestingly, both metastatic cell lines were significantly less sensitive to radiation at both 4 and 8 Gy (*p* <0.01, one way ANOVA, Tukey's *post‐hoc* test).

## Discussion

We have developed a rodent model of an aggressive extremity fibrosarcoma to allow the development and assessment of novel treatment combinations to optimise tumour control. This model closely resembles the clinical scenario seen in many patients with advanced limb sarcomas after treatment with standard ILP, where an initial local response is followed by local disease progression that may occur before the development of metastatic disease. Using this model, we have previously shown that the addition of oncolytic virotherapy to the chemotherapeutic agents currently used in ILP has a synergistic effect.^9^ However, long‐term local control of the limb sarcoma was not achieved. We have now modified this treatment strategy to further improve the efficacy of this approach and hopefully produce long‐term complete responses in the limb. With this aim in mind, we used oncolytic virotherapy together with standard ILP chemotherapy but in combination with surgical resection of the tumour and radiation. The addition of surgical resection and radiotherapy to ILP is clinically relevant as ILP has been shown to be an effective neoadjuvant therapy prior to surgical resection of large, locally advanced ESTS as a means of allowing limb salvage in what would be considered normally an irresectable tumour. Furthermore, postoperative radiotherapy may also be used if the histological response to ILP is inadequate.^4^, ^14^, ^21^, ^22^

After demonstrating an additive effect *in vitro*, combinations of oncolytic virotherapy delivered by ILP with radiation were evaluated in our *in vivo* model. Although the combination of radiation with standard ILP chemotherapy (melphalan and TNFα) improved survival when compared with standard ILP alone, this combination was no more effective than triple therapy ILP (melphalan, TNFα and OV). Furthermore, the addition of radiation to triple therapy ILP conferred no further survival benefit. In the absence of a surgical resection, local disease control in this model could not be achieved. This scenario closely resembles that seen in clinical practice. Without surgical resection, the majority of patients with ESTS treated by ILP develop disease progression within 12 months.^7^ At this point, further disease progression is extremely challenging to control with any treatment modality and many patients proceed to a palliative amputation.^5^, ^7^

To explore the benefit of surgical resection alongside these treatments we used two *in vivo* protocols combining triple therapy ILP and radiation with surgery. In these protocols all surgical resections were marginal resections, performed on the surface of these large tumours and would be classified clinically as R1 resections leaving likely microscopic residual disease *in situ*.

The first protocol was designed to maximise the additive effects of radiation and oncolytic virotherapy and to allow time for a viral‐mediated anti‐tumour immune response to develop and surgery was deferred until after radiotherapy had been given. The second protocol was designed to mirror the normal clinical practice of neoadjuvant ILP followed by a surgical resection and radiotherapy was administered postoperatively to microscopic residual disease. The timing of surgery was found to be critical in producing on‐going complete remissions in the limb, with local disease control only being achieved with early surgical intervention. There may be several reasons for this. Live, replication competent virus was retrieved from five of the six tumours resected 72 hr after triple therapy ILP. Resecting the tumour at this time point may have enabled the residual virus to control any remaining microscopic disease, with its therapeutic effect enhanced by the addition of radiation. A further hypothesis is that with time, a more heterogeneous tumour cell population develops, with increased treatment resistance. Early surgical excision may interrupt this development and improve outcome.

The curative treatment protocol developed in our model was intensive, although it was well tolerated with no increase in wound complications or functional morbidity when compared with other cohorts. In clinical practice, the combination of ILP, surgery and postoperative radiotherapy has been shown to be associated with poorer wound healing and long‐term functional morbidity in up to two‐thirds of patients.^23^, ^24^ In the presence of an adequate response to ILP, as determined histologically by the percentage of necrosis of the specimen, adjuvant radiotherapy has not been shown to be of further benefit in securing local disease control.^25^ Therefore, in order to balance local control with functional morbidity, adjuvant radiotherapy is now reserved for those patients with an inadequate response to ILP. Although the data from our *in vivo* experiments are encouraging, whether a modified protocol with the addition of an oncolytic virus will be as tolerable and as efficacious in the clinic requires clarification in a phase I clinical trial.

ESTS comprise a heterogeneous mix of histological subtypes. A limitation to this study is that only one cell line, BN175, could be used for *in vivo* experimentation. In order to more accurately study the effects of virotherapy, an immune competent model is desirable and precludes the use of human xenografts. Although a mouse model of ILP has been described, this was not found to be reproducible by our team.^26^ As such, *in vivo* experiments were limited to the BN175 cell line, as it alone was syngeneic in rats. *In vitro* experiments provided encouraging data suggesting that this combination treatment has the potential for clinical translation. However, these experiments were limited to fibrosarcoma and liposarcoma lines and whether this approach will be as effective across a range of histological subtypes remains to be seen.

With improved local disease control, the metastatic potential of this tumour model was revealed. This development provides a platform for future work aimed at interrupting the metastatic pathway in this model. Oncolytic virotherapy is increasingly considered as a form of immunotherapy. The ability to promote an anti‐tumour immune response has been demonstrated with several oncolytic viruses including vaccinia, herpes simplex virus, reovirus, parvovirus and myxoma virus.^10^, ^27^, ^28^, ^29^, ^30^ Furthermore, there is evidence to suggest that immune activation rather than viral replication or oncolysis is key to the tumouricidal effects of oncolytic virotherapy.^31^ Although oncolytic vaccinia was unable to prevent metastatic disease in our model, future work will be directed at combining oncolytic virotherapy with other immunomodulatory adjuvant therapies with the aim of preventing and treating metastases. If their potential were realised, these combinations may provide a means of engendering a tumour‐specific immune response prior to the resection of a tumour to safeguard against both local and distant recurrence.

## Supporting information

Supporting Information
