# Supplementary figures and images for: Isolated limb perfusion with biochemotherapy and oncolytic virotherapy combines with radiotherapy and surgery to overcome treatment resistance in an animal model of extremity soft tissue sarcoma
Source: Int J Cancer. 2016 May 24;139(6):1414–22. doi: 10.1002/ijc.30162 (PMC5082541; doi:10.1002/ijc.30162)

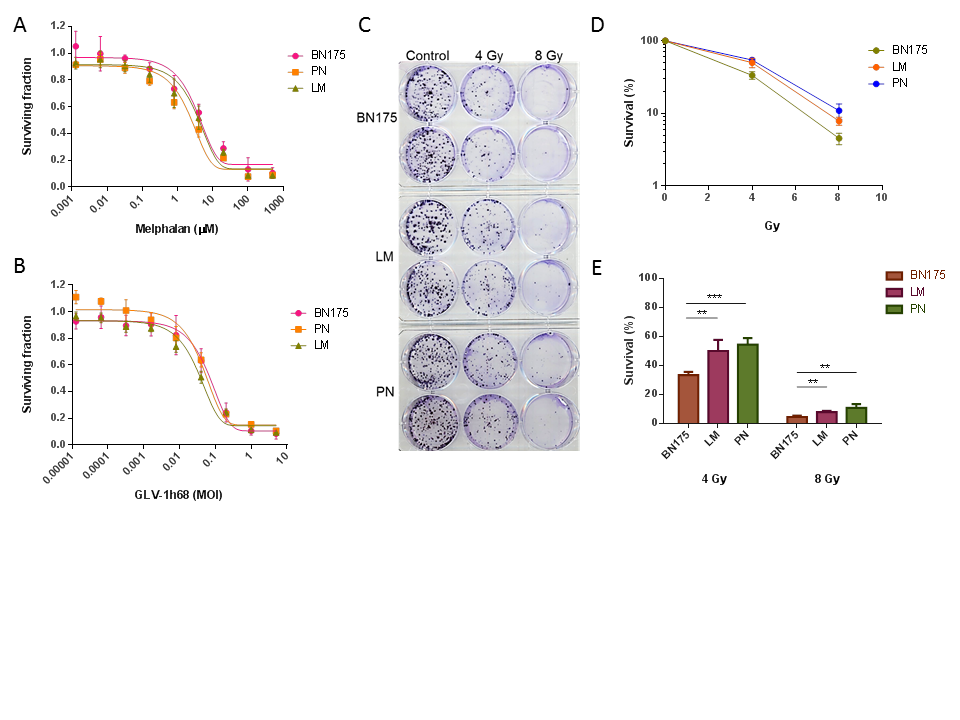

Supplement: Supplementary file 1 — Supporting Information [file IJC-139-1414-s001.TIF]
